# Primary health care and sustainable development goals

**DOI:** 10.2471/BLT.19.245613

**Published:** 2020-09-03

**Authors:** Somtanuek Chotchoungchatchai, Aniqa Islam Marshall, Woranan Witthayapipopsakul, Warisa Panichkriangkrai, Walaiporn Patcharanarumol, Viroj Tangcharoensathien

**Affiliations:** a International Health Policy Program, Ministry of Public Health, Tivanond Road, 11000 Nonthaburi, Thailand.

## Abstract

We examine the potential and limitations of primary health care in contributing to the achievement of the health-related sustainable development goals (SDGs), and recommend policies to enable a functioning primary health-care system. Governments have recently reaffirmed their commitment to the SDGs through the 2018 Declaration of Astana, which redefines the three functions of primary health care as: service provision, multisectoral actions and the empowerment of citizens. In other words, the health-related SDGs cannot be achieved by the provision of health-care services alone. Some health issues are related to environment, necessitating joint efforts between local, national and international partners; other issues require public awareness (health literacy) of preventable illnesses. However, the provision of primary health care, and hence achievement of the SDGs, is hampered by several issues. First, inadequate government spending on health is exacerbated by the small proportions allocated to primary health care. Second, the shortage and maldistribution of the health workforce, and chronic absenteeism in some countries, has led to a situation in which staffing levels are inversely related to poverty and need. Third, the health workforce is not trained in multisectoral actions, and already experiences workloads of an overwhelming nature. Finally, health illiteracy is common among the population, even in developed countries. We recommend that governments increase spending on health and primary health care, implement interventions to retain the rural health workforce, and update the pre-service training curricula of personnel to include skills in multisectoral collaboration and enhanced community engagement.

## Résumé

Dans le présent document, nous étudions le potentiel et les limites des soins de santé primaires en matière de réalisation des objectifs de développement durable (ODD) liés à la santé. Nous formulons également des recommandations politiques pour l'instauration d'un système de soins de santé primaires efficace. Les gouvernements ont récemment réaffirmé leur engagement envers les ODD en 2018 par le biais de la Déclaration d'Astana, qui redéfinit les trois fonctions des soins de santé primaires comme suit: fourniture de services, actions multidisciplinaires et implication des citoyens. En d'autres termes, les ODD liés à la santé ne peuvent être atteints uniquement en proposant des services médicaux. Certains problèmes de santé sont inhérents à l'environnement et requièrent les efforts conjoints des partenaires locaux, nationaux et internationaux; d'autres nécessitent de sensibiliser le public (médecine préventive) aux maladies évitables. Cependant, la fourniture de soins de santé primaires, et par conséquent la réalisation des ODD, rencontre de nombreux obstacles. Tout d'abord, les dépenses inadéquates des gouvernements en matière de santé sont aggravées par le faible pourcentage octroyé aux soins de santé primaires. Ensuite, la pénurie et les inégalités de répartition des professionnels de la santé ainsi que l'absentéisme chronique dans certains pays ont débouché sur une situation où le niveau des effectifs est inversement proportionnel au niveau de pauvreté et aux besoins. Par ailleurs, le personnel soignant n'est pas formé aux actions multidisciplinaires et subit déjà une charge de travail écrasante. Et enfin, la méconnaissance des bases sanitaires est fréquente au sein de la population, même dans les pays développés. Nous conseillons aux gouvernements d'accroître leurs dépenses en soins de santé et soins de santé primaires, d'intervenir pour encourager les soignants à rester dans les régions rurales, et de mettre à jour les programmes de formation initiale du personnel pour y inclure des compétences en collaboration multidisciplinaire et en amélioration de l'engagement communautaire.

## Resumen

Se analizan las posibilidades y las limitaciones de la atención primaria de salud para contribuir al logro de los objetivos de desarrollo sostenible (los ODS) relacionados con la salud, y se recomiendan políticas que permitan el funcionamiento del sistema de atención primaria de salud. Recientemente, los gobiernos reiteraron su compromiso con los ODS en la Declaración de Astaná de 2018, en la que se redefinen las tres funciones de la atención primaria de salud, a saber: la prestación de servicios, las medidas multisectoriales y una mayor participación de los ciudadanos. Es decir, los ODS relacionados con la salud no se pueden cumplir tan solo con la prestación de servicios de atención de la salud. Algunos temas de salud están relacionados con el medio ambiente, lo que requiere esfuerzos conjuntos entre los asociados locales, nacionales e internacionales; otros temas requieren la concienciación del público (conocimientos sobre la salud) acerca de las enfermedades que se pueden evitar. Sin embargo, la prestación de atención primaria de salud, y por consiguiente el logro de los ODS, presenta diversas dificultades. En primer lugar, el gasto público inadecuado en salud empeora debido a los porcentajes tan reducidos que se asignan a la atención primaria de salud. En segundo lugar, la escasez y la mala distribución del personal sanitario, así como el absentismo crónico en algunos países, han creado una situación en la que los niveles de personal están relacionados de manera inversa con la pobreza y la necesidad. En tercer lugar, el personal sanitario no está capacitado para emprender medidas multisectoriales, además de que ya tiene una carga de trabajo abrumadora. Por último, la falta de conocimientos sobre salud es común entre la población, incluso en los países desarrollados. Se recomienda a los gobiernos que aumenten el gasto en salud y en atención primaria de la salud, que implementen intervenciones para fidelizar al personal sanitario de las zonas rurales y que actualicen los programas de capacitación del personal previa a la prestación de servicios para integrar las habilidades en la colaboración multisectorial y el aumento de la participación de la comunidad.

## ملخص

نحن نقوم بدراسة إمكانات وحدود الرعاية الصحية الأولية في المساهمة لتحقيق أهداف التنمية المستدامة المتعلقة بالصحة (SDG)، ونوصي بسياسات لتمكين نظام فعال للرعاية الصحية الأولية. أعادت الحكومات مؤخرًا التأكيد على التزامها بأهداف التنمية المستدامة من خلال إعلان أستانا (Declaration of Astana) لعام 2018، والذي يعيد تعريف الوظائف الثلاث للرعاية الصحية الأولية على النحو التالي: تقديم الخدمات، والإجراءات متعددة القطاعات، وتمكين المواطنين. وبعبارة أخرى، لا يمكن تحقيق أهداف التنمية المستدامة المتعلقة بالصحة عن طريق توفير خدمات الرعاية الصحية وحدها. تتعلق بعض القضايا الصحية بالبيئة، مما يتطلب تضافر الجهود المبذولة بين الشركاء المحليين والوطنيين والدوليين؛ بينما تتطلب قضايا أخرى التوعية العامة (الوعي الصحي) بالأمراض التي يمكن الوقاية منها. ومع ذلك، فإن توفير الرعاية الصحية الأولية، ومن ثم تحقيق أهداف التنمية المستدامة، تعوقه العديد من القضايا. أولاً، يظهر الإنفاق الحكومي غير الكافي على الصحة بشكل واضح بسبب النسب الصغيرة المخصصة للرعاية الصحية الأولية. ثانيًا، أدى النقص في القوى العاملة الصحية وسوء توزيعها، والتغيب المتواصل عن العمل في بعض البلدان، إلى موقف ترتبط فيه مستويات التوظيف ارتباطًا عكسيًا مع الفقر والحاجة. ثالثًا، القوى العاملة الصحية ليست مدربة على الإجراءات متعددة القطاعات، وهي تواجه بالفعل أعباء عمل ذات طبيعة مهلكة. وأخيرًا، فإن الأمية الصحية شائعة بين السكان، حتى في البلدان المتقدمة. نحن نوصي الحكومات بأن تزيد من الإنفاق على الصحة والرعاية الصحية الأولية، وتنفيذ التدخلات للحفاظ بالقوى العاملة الصحية الريفية، وتحديث مناهج التدريب للموظفين قبل الخدمة لتشمل مهارات التعاون متعدد القطاعات، والمشاركة المجتمعية المعززة.

## 摘要

我们调查了初级卫生保健在促进实现卫生可持续发展目标（SDGs）方面的潜力和局限性，并提出了建议措施，帮助初级卫生保健体系正常运转。各国政府最近通过 2018 年《阿斯塔纳宣言》重申了对可持续发展目标做出的承诺，该宣言重新定义了初级卫生保健的三项职责：提供服务、多部门合作和公民赋权。换言之，仅靠提供卫生保健服务无法实现卫生可持续发展目标。一些卫生问题与环境有关，因此需要地方、国家和国际上的共同努力；其他问题需要民众了解可预防的疾病（健康素养）。但有几个问题妨碍了初级卫生保健的供应，从而影响可持续发展目标的实现。首先，分配给初级卫生保健方面的资金少，从而导致政府对卫生的支出大大下降。其次，医务人员不足和分布不均，以及一些国家的长期不作为导致人员配置水平与贫困和需求成反比。第三，医务人员未接受过多部门实践培训，便承受了巨大的工作量。最后，即使在发达国家，文盲现象仍普遍存在。我们建议政府加大对卫生和初级卫生保健的支出，采取干预措施以留住农村地区的医务人员，并更新人员岗前培训课程，帮助其掌握多部门合作和增强社区参与的技能。

## Резюме

Авторы изучают потенциал и ограничения первичной медико-санитарной помощи в плане содействия достижению целей устойчивого развития (ЦУР), связанных со здоровьем, и рекомендуют политики, которые позволяют создать работающую систему первичной медико-санитарной помощи. Правительства недавно подтвердили свою приверженность ЦУР в Астанинской декларации от 2018 года, которая заново определяет три функции первичной медико-санитарной помощи, а именно: предоставление услуг, межсекторальную деятельность и расширение прав и возможностей граждан. Другими словами, связанные со здоровьем ЦУР не могут быть достигнуты только за счет предоставления медицинских услуг. Некоторые вопросы в сфере здравоохранения связаны с окружающей средой, что требует совместных усилий местных, национальных и международных партнеров. Другие вопросы требуют осведомленности общественности (санитарной грамотности) о болезнях, которые можно предотвратить. Однако предоставлению первичной медико-санитарной помощи и, следовательно, достижению ЦУР препятствуют несколько проблем. Во-первых, недостаточные государственные расходы на здравоохранение усугубляются малой долей средств, выделяемых на первичную медико-санитарную помощь. Во-вторых, нехватка и неправильное распределение кадровых ресурсов здравоохранения, а также хроническое уклонение от работы в некоторых странах привели к ситуации, в которой уровни укомплектования кадрами обратно пропорциональны бедности и потребностям. В-третьих, кадровые ресурсы здравоохранения не обучены межсекторальной деятельности и уже испытывают огромные нагрузки. И наконец, неграмотность в сфере здравоохранения распространена среди населения даже в развитых странах. Авторы рекомендуют правительствам увеличить расходы на здравоохранение и первичную медико-санитарную помощь, внедрить меры по сохранению кадров здравоохранения в сельских районах и обновить программы предварительного обучения персонала, включив в них навыки межсекторального сотрудничества и методы привлечения населения к более активному участию.

## Introduction

The concept of primary health care has been at the centre of the health development agenda for many decades, originally inspired by the 1948 World Health Organization (WHO) constitution^1^ and later the focus of the Alma-Ata International Conference on Primary Health Care in September 1978.^2^^,^^3^ Primary health care is a whole-society approach to health, and aims to attain the highest possible level and distribution of health and well-being by providing an accessible and wide range of services, including: health promotion; disease prevention, treatment and rehabilitation; and palliative care.^4^ As the first contact between the population and the health-care system, primary care is essential for integrated personal health care, public health function and ongoing referrals to hospital services. However, the provision of primary care services is only one part of the broader primary health-care concept;^5^ to address the determinants of health, the implementation of a primary health-care concept must be accompanied by multisectoral actions and the empowerment of the population.^6^

Of the 17 sustainable development goals (SDGs) adopted at the Seventieth session of the United Nations (UN) General Assembly in 2015,^7^ to be achieved by 2030,^8^ the third (SDG 3, to “ensure healthy lives and promote well-being for all at all ages”) is specific to health. SDG 3 includes the provision of universal health coverage (UHC; SDG 3.8), which aims to provide access to good-quality health services for all, without financial hardship. Other SDGs (e.g. on hunger; gender equality; clean water and sanitation; affordable and clean energy; sustainable cities and communities; climate action; and peace, justice and strong institutions) also contribute indirectly to the attainment of health. 

To maintain momentum in the provision of primary health care and UHC, governments have recently reaffirmed their commitments to the SDGs through the 2018 Astana Declaration^9^ and the 2019 UN High-Level Meeting on UHC.^10^ The Astana Declaration redefined the three main functions of primary health care as: (i) meeting the health needs of a population through the provision of a comprehensive range of promotive, protective, preventive, curative, rehabilitative and palliative health-care services throughout the life course; (ii) systematically addressing the broader determinants of health including social, economic and environmental contexts through evidence-informed public policies and multisectoral action; and (iii) empowering individuals, families and communities to optimize their health, and supporting people such as self-carers and caregivers as co-developers of health and social services.^11^

We examine the potential and limitations of primary health care in contributing to health-related SDGs, identify barriers to the provision of primary health care, and recommend policies for implementation to enable a fully functioning primary health-care service.

## Comparison with UHC

**Fig. 1:**
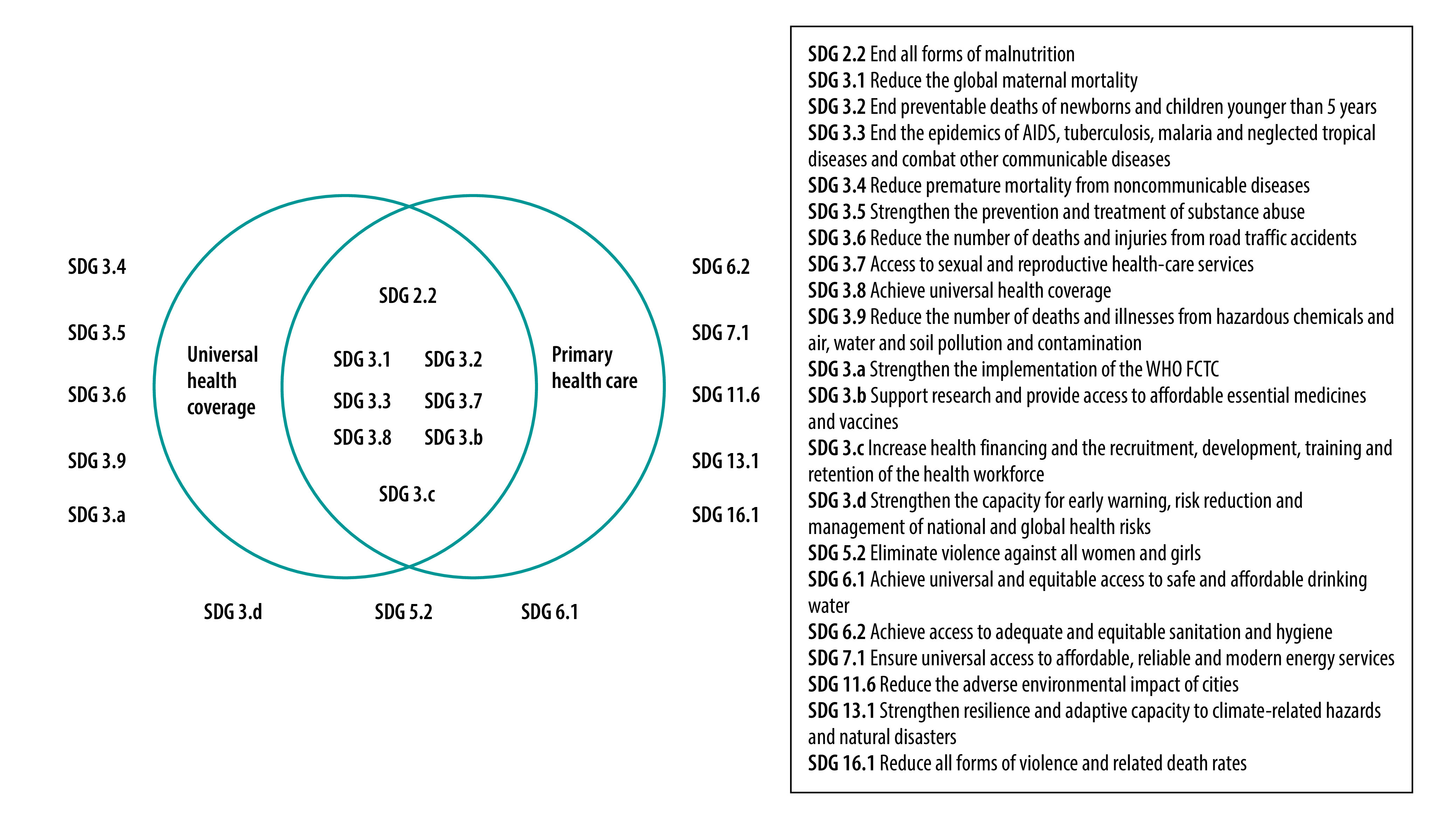
The contribution of primary health care and universal health coverage to the achievement of health-related sustainable development goals

AIDS: acquired immunodeficiency syndrome; FCTC: Framework Convention on Tobacco Control; SDG: sustainable development goal; WHO: World Health Organization.

Note: The SDGs in the intersection of the two circles can be addressed by the synergies of universal health coverage and primary health care, while SDGs lying outside the two circles require multisectoral action and citizen empowerment.

The provision of a health service is the main function of both primary health care and UHC;^12^ we indicate in Fig. 1 where these two systems overlap in terms of achieving the SDGs. Both primary health care and UHC advocate for the whole continuum of required health services, and not just for first-contact or ambulatory health services. For example, the provision of antiretroviral, anti-tuberculosis and antimalarial drugs (SDG 3.3) requires a higher level of clinical competency, that is, as provided by secondary- or tertiary-level facilities, beyond the scope of first-contact primary care. Finally, while the primary-care system is focused on the provision of good-quality and coverage of essential health services (SDG 3.8.1), it does not address the avoidance of financial hardship in using these services; however, UHC includes protection from financial risk (SDG 3.8.2).

## Potential

The health-related SDGs cannot be achieved by the provision of high-quality primary health-care services alone. Some health issues are related to a person’s environment, necessitating joint efforts between sectors from local to national and international development partners; other issues require public awareness (health literacy) of preventable illnesses and the provision of supportive environments. We describe in the following, and depict in Fig. 1, how primary health care as defined by the Astana Declaration (i.e. advocating for multisectoral actions and the empowerment of citizens) contributes to the achievement of both health-specific SDG 3 and the other health-related SDGs. 

### SDG 3

The goal of ending the prevalence of neglected tropical diseases (SDG 3.3) is an example of where collective efforts are required. Cost-effective interventions are available for as little as 3 United States dollars (US$) per disability-adjusted life year (DALY) averted.^13^ However, multisectoral actions to address poverty, control disease vectors and the environment, and improve access to clean water and sanitation are key components of neglected tropical disease programmes. For example, the elimination of trachoma has been a collective effort beyond national boundaries between development partners, the private sector and governments.^14^

The achievement of SDG 3.4 on noncommunicable diseases also requires multisectoral actions to address the determinants of health. To some extent, primary health care and UHC can secure the availability and affordability of essential medicines for the treatment of noncommunicable diseases, such as insulin and metformin for diabetes, diuretics for hypertension and statins for hyperlipidaemia. However, the provision of access to essential medicines also requires multisectoral collaborations at the national level to exercise Trade-Related Aspects of Intellectual Property Rights flexibilities,^15^^,^^16^ as envisioned by the Doha Declaration. In addition, empowering individuals and families by improving health literacy can encourage lifestyle changes and the subsequent prevention of noncommunicable diseases. Population-based interventions addressing the distal determinants of health risk (e.g. increasing taxation on tobacco, alcohol and unhealthy foods; improving physical and social environments, conducive to active lifestyles) yield higher health gains than addressing the proximal determinants through the screening and treatment of individual patients at the primary-care level.^17^ In terms of addressing tobacco use, Table 1 demonstrates the higher potential and capacity of multisectoral actions compared with those of the primary health-care system. 

**Table 1:** Interventions to address tobacco use: what multisectoral actions can achieve compared with primary health care

Interventions^17^	Multisectoral actions	Primary health care
**Best buys: effective interventions^a^**		
Increase excise taxes and prices on tobacco products	Legislative and regulatory measures by ministry of finance; high-level political leadership in the context of political interference by tobacco industry;^18^ addressing the legal threats of the tobacco industry;^19^ civil society role in monitoring interference;^20^ and government application of tobacco strategic litigation^19^	Very limited role and capacity
Implement plain, standardized packaging and/or large graphic health warnings on all tobacco packages	Legislative and regulatory measures, with penalty for non-adherence	Very limited role and capacity
Enact and enforce comprehensive bans on tobacco advertising, promotion and sponsorship	Legislative measures, surveillance and legal sanction of non-adherence	Very limited role and capacity
Eliminate exposure to second-hand tobacco smoke in all indoor workplaces, public places and on public transport	Public and private sector (including civil society organizations) support of smoke-free indoor workplaces and public transport, and monitor non-adherence	Create public awareness and social demand for tobacco-free environment; surveillance of non-adherence
Implement effective mass media campaigns that educate the public about the harms of tobacco use and second-hand smoke	Public and private sector support of media campaigns	Support community-based public awareness campaigns
**Effective interventions^b^**		
Provide support, advice and national toll-free helpline services for tobacco cessation	Ministry of health and civil society organizations support tobacco cessation services	Key role in providing counselling service
**WHO-recommended interventions^c^**		
Implement measures to minimize illicit trade in tobacco products	Active surveillance and law enforcement by customs departments	No role
Ban cross-border advertising, including using modern means of communication	Active monitoring by departments of information and communication technology, and by digital experts	Support regulatory bodies in surveillance of non-adherence
Provide mobile-phone-based tobacco cessation services	Ministry of health and civil society organizations support tobacco cessation services	Key role in providing counselling service

WHO: World Health Organization.

^a^ The cost was less than 100 international dollars per disability-adjusted life year (DALY) averted in low- and middle-income countries.

^b^ Cost–effectiveness ratio of more than 100 international dollars per DALY averted in low- and middle-income countries.

^c^ No cost–effectiveness data available.

Preventing the harmful use of alcohol (SDG 3.5) also requires best-buy interventions, defined as interventions costing at most 100 international dollars per DALY averted in low- and middle-income countries.^17^ Such interventions include controlling availability through legal age limits, service hours and number of sales outlets, increasing tax and retail price, and prohibiting advertisements and market promotion. Effective control of substances requires a strong collaboration between health and law enforcement officers, framed by good governance. These actions require policy coherence across government sectors and are the remit of national-level actors, notably finance ministries and regulatory bodies for sales and promotion of these harmful products. 

Finally, the provision of good-quality health care on its own makes a minimal contribution to achieving SDG 3.6, halving the number of global deaths and injuries from road traffic accidents. Primary health care does have a role in pre-hospital service, referral to a hospital-based accident and emergency unit, and post-crash responses such as minimizing time between road traffic incidents and the provision of first response emergency care. However, other primary prevention methods, such as (i) technical standards of new and existing roads, taking into account international safety standards; (ii) the adherence of new and used vehicles to safety standards; (iii) the use of seat belts, child restraint systems and helmets; (iv) the designation and regulation of speed limits; (v) legislation and enforcement of blood alcohol content and use of mobile phones while driving; and (vi) driving time and rest periods for professional public transport drivers, are the responsibility of other sectors and regulatory bodies.^21^

### Other health-related SDGs

Progress towards other health-related SDGs also requires a multisectoral approach. Strong governance is required, as all actors must articulate their interests, exercise their rights and obligations, and mediate their differences.^22^

For example, to end hunger (SDG 2), primary health care can support community-based therapeutic care using ready-to-use, therapeutic foods for severe acute malnutrition;^23^ however, a more sustainable solution lies in the adoption of agricultural policies that aim to double the productivity and income of small-scale food producers. 

To achieve SDG 11 on the creation of sustainable cities, primary health care can support the monitoring of air pollution, issue health warnings when particulate matter of diameter less than 2.5 μm in the air exceeds limits, and advocate for the reduction of indoor pollution through the use of clean energy.^24^ However, sustainable solutions can only be achieved through policies that address industrial, residential and vehicular sources of pollution. 

## Limitations

We have identified four underlying factors currently impeding the full implementation of the three primary health-care functions, and hence achievement of the SDGs. The factors reviewed here, in particular inadequate funding, primary health-care workforce shortage and insufficient multisectoral actions, were also highlighted in an online survey of 50 WHO Member States conducted by the WHO Evaluation Office.^25^

### Inadequate funding

**Fig. 2:**
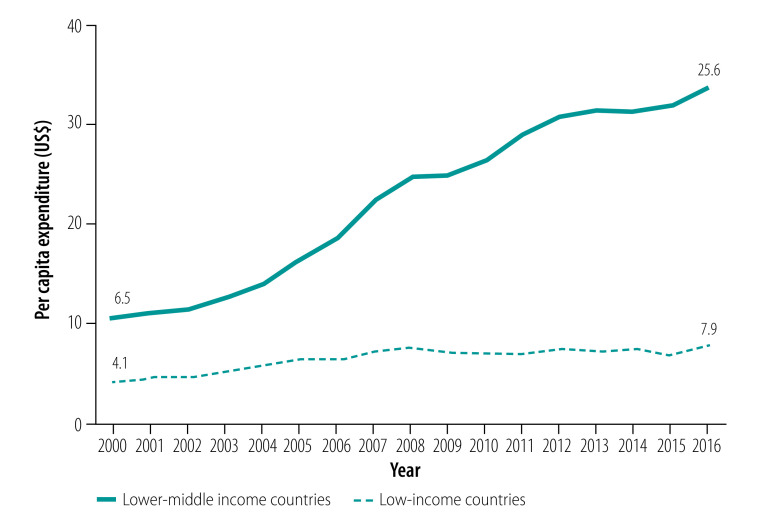
Domestic general government health expenditure per capita, 2000–2016

Inadequate spending on primary health care undermines the development of related infrastructure, including the capacity for disease prevention and health promotion. The average government health expenditure per capita increased from US$ 4.1 to US$ 7.9 in low-income countries, and from US$ 6.5 to US$ 25.6 in lower-middle-income countries, between 2000 and 2016.^26^ However, a minimum government health spending of US$ 91 and US$ 89 per capita per year, in low-income and lower-middle-income countries, respectively, is required by 2030 to achieve SDG 3 (Fig. 2).^27^

US$: United States dollars.

Source: World Bank.^26^

As well as spending levels on health being too low, they can also be biased towards curative services and hospital care; any rise in health spending is often driven by medical technologies, particularly in the hospital sector.^28^ In high income countries, the hospital sector consumed 38% of total current health spending in 2016 (average of 28 European Member States)^29^ while spending on primary care was only 14% of total health spending (average of 22 Organisation for Economic Co-operation and Development, OECD, Member States).^30^

This problem of underfunding is impounded by the lack of a credible system to properly monitor primary health-care expenditure. The National Health Account, guided by the 2011 OECD Systems of Health Account, does not provide an estimate of the total spending on primary health care as a percentage of current health expenditure;^31^ however, a study in 36 low- and middle-income countries showed an interquartile range of primary health-care expenditure of US$ 15–60 per capita, or 31–88% of current health expenditure.^32^ These insufficient data hamper national and global monitoring of adherence to the 2019 UN General Assembly resolution 74/2, for which an additional investment in primary health care of 1% of gross domestic product was agreed to by UN Member States.^10^

### Workforce shortage

Provision of a comprehensive range of health services is hampered by a health workforce shortage and maldistribution, something that has always been a chronic problem for low- and middle-income countries. In 2006, the World Health Report identified 57 Member States with a density of health workers below the benchmark of 22.8 doctors, nurses and midwives per 10 000 population (the minimum required for 80% of births to be attended by a skilled health professional); the most significant shortfalls occurred in sub-Saharan Africa.^33^ Our primary analysis revealed no improvement since 2006; in 2019, 62 of 194 WHO Member States (32%) reported a health worker density of less than the above-mentioned benchmark.^34^

**Fig. 3:**
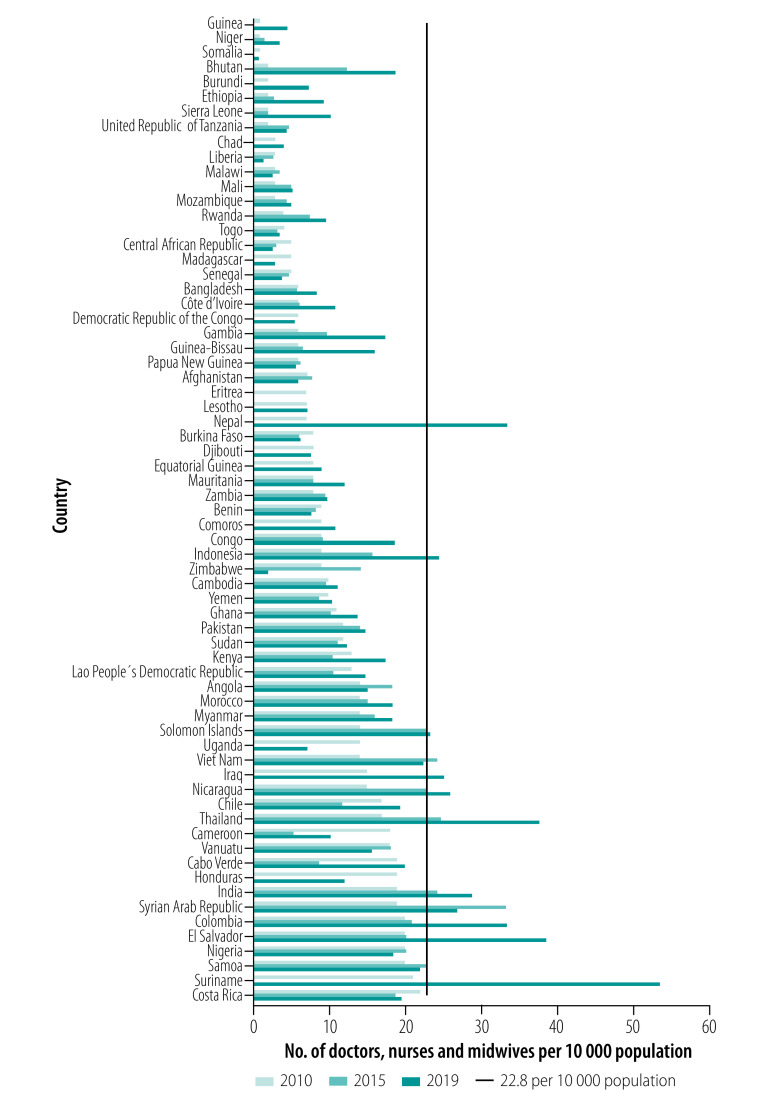
Changes in health workforce density in 67 countries, 2010–2019

Note: We included countries with less than 22.8 doctors, nurses and midwives per 10 000 population in 2010: no data were available for some countries for the year 2015.

Source: *World Health Statistics 2010*,^35^ *2015*^36^ and *2019*.^34^

Analysis by World Health Statistics, depicted in Fig. 3, indicates a gloomy and unchanging situation in health workforce density from 2010 to 2019; of the 67 countries that fell below the benchmark in 2010, 21 countries (31%) showed no progress by 2019, while 35 countries (52%) had progressed but remained below the threshold. Only 11 countries (16%) had progressed to an adequate density of health workers. 

The basic health worker density needed to achieve the SDGs has been reported by the WHO as 44.5 per 10 000 population,^37^ meaning that almost half of the Member States (96/194; 49%) had an insufficient health workforce in 2019. With this critical shortage, exacerbated by poor distribution of rural primary care and a lack of necessary skills, the commitments of the Astana Declaration appear to be out of reach for the immediate future.

Absenteeism of frontline health workers is not uncommon in many Asian and African countries, and the root cause of a poorly functioning primary care system and the lack of population trust in service quality. A survey of Indonesian community health centre staff, including 20 doctors, 61 midwives and 76 paramedics, reported an overall absenteeism rate of 23%.^38^ A multicountry study in developing countries reported higher rates of absenteeism in higher-level cadres, including those with more opportunity for private practice.^39^

A multicountry study in five African countries highlighted that the minority of the health workforce are employed in primary health care, with greatest shortages in rural areas.^40^ This finding illustrates what is referred to as the “inverse primary health care law,” where staffing levels are inversely related to poverty and level of need. According to 66 migrant health personnel from sub-Saharan African countries, this inverse primary health care law is explained by poor working environments, difficult living conditions and a perceived inferior career path, leading to high levels of out-migration.^41^ In the United Kingdom of Great Britain and Northern Ireland, a high-income country, the inverse primary health care law also exist; citizens in the most deprived areas have higher levels of multimorbidity and are deprived access to high-quality primary health care.^42^

### Limited multisectoral action

The capacity for multisectoral action at local communities as demanded by the Astana Declaration is often limited. Despite its potential to significantly address many social and economic determinants of health, multisectoral collaboration is not generally or adequately practised. Health personnel, particularly at the primary-care level, are not trained in multisectoral skills.^43^ The workload of public health and curative services is often overwhelming, reducing the likelihood of interaction with actors outside the health sector. Multisectoral collaborations also require social credibility and convening power, which is too demanding for facilities with few or young primary health-care staff and a high turnover rate. 

Another problem is that multisectoral actions at the community level, such as legislative and regulatory tools to apply and scale-up best-buy interventions, have limited impact on the distal determinants of health.^17^ While proximal determinants of health (e.g. lifestyle and socioeconomic status) can be addressed by the primary health-care system, distal determinants (e.g. legal and cultural) can be tackled more effectively through national-level mechanisms including legislation, regulation and enforcement. Effective multisectoral action therefore requires political commitment, as well as a balance between competition and collaboration among government agencies.^44^

### Inadequate empowerment

Finally, the primary health-care function of empowering citizens provides another challenge. Although the primary health-care setting has the comparative advantage of its proximity to the people and community, strengthening health literacy requires actions beyond the microlevel. The experience of high-income countries has shown that health literacy education or partnerships between adult education programmes and primary health care providers can support a wide range of actions that improve health and well-being.^45^

The importance and benefits of health literacy are clear; however, the WHO Regional Office for Europe provided evidence of limited health literacy as recently as 2013. In the European Health Literacy Survey of 7795 people from eight countries, an average of 12% of the respondents demonstrated an inadequate general health literacy (ranging from 2% out of 993 respondents in the Netherlands and 27% out of 925 respondents in Bulgaria), and an average of 35% with a limited health literacy (as measured by those with inadequate literacy plus those with problematic literacy; ranging from 29% for the Netherlands to 62% in Bulgaria).^46^^,^^47^

A recent systematic review reported a lack of institutional support, data and resources required for community and citizen empowerment and for participatory and deliberative approaches in health policy-making processes in sub-Saharan Africa.^48^ Despite the existence of participatory health councils in 99% (1972/2000) of all Brazilian cities, there still exists a lack of full citizen and community empowerment in health policy decision-making.^49^^,^^50^

## Recommendations

To overcome the issues affecting the successful implementation of the primary health-care functions, and hence achievement of UHC and other health-related SDGs, we recommend that governments in low- and middle-income countries take the following actions. 

First, governments must significantly increase their financial commitment to the health sector, but also increase the proportion of this spending in the area of primary health care by streamlining hospital spending. 

Second, we recommend that governments implement programmes focused on the effective retention of the health workforce in rural primary care services.^51^ Bundle interventions, such as providing integrated training and career pathways, financial and non-financial incentives, and safe and supportive work environments, as well as meeting the needs of both individual health-care workers and those of their families, can be more effective than any single intervention.^52^

Third, we recommend that pre-service training curricula of primary health-care personnel are updated to ensure that, as well as skills in delivering clinical services, staff have the opportunity to acquire (i) awareness of the importance of public health interventions and (ii) competence in collaborating with communities and other sectors. Further, local- and national-level multisectoral actions should be synergized to address the determinants of health through the application of best-buy interventions. For example, the local-level law enforcement of smoke-free public spaces, alcohol breath testing^53^ and maximum driving speeds^54^ can be synergized with national-level interventions such as increased tax, controlled tobacco marketing, and legislation on alcohol and vehicle speed limits. 

Finally, to improve the health literacy of populations, we recommend that the skills of primary health-care workers be enhanced to include the establishment of official platforms that could support community engagement in multilevel decision-making in health care. 
